# Neutron Reflectometry Reveals Diffusion in Contrast-Matched Brush Particle Bilayers

**DOI:** 10.1021/acsmacrolett.6c00109

**Published:** 2026-04-06

**Authors:** Jirameth Tarnsangpradit, Yuqi Zhao, Hanshu Wu, Rongguan Yin, Hanyu Wang, Akhtar Gul, Alamgir Karim, Krzysztof Matyjaszewski, Michael R. Bockstaller

**Affiliations:** † Department of Material Science and Engineering, 6612 Carnegie Mellon University, 5000 Forbes Avenue, Pittsburgh, Pennsylvania 15213, United States; ‡ Department of Chemistry, 6612 Carnegie Mellon University, 4400 Fifth Avenue, Pittsburgh, Pennsylvania 15213, United States; § Center for Nanophase Materials Sciences, 6146 Oak Ridge National Laboratory, Oak Ridge, Tennessee 37831, United States; ∥ Department of Chemical and Biomolecular Engineering, 14743 University of Houston, Houston, Texas 77204, United States

## Abstract

A material system for performing layer-spread experiments on brush particle bilayers is presented and used to determine the diffusion constant of brush particles in the melt state. Selective deuteration of the core and shell of organo-silica nanoparticles grafted with poly­(methyl methacrylate) was used to match the scattering length density of the core and the polymer canopy layer. This subdued the scattering of particle cores (i.e., formfactor scattering) and enabled the analysis of the interdiffusion kinetics using neutron reflectivity. For low molecular grafts, i.e., grafts with a molecular weight below the entanglement limit, the interdiffusion kinetics revealed both a sub- and Fickian diffusion regime. The former was attributed to the local dynamics that was constrained by the slow-moving cores of neighboring brush particles that acted as long-lived physical cross-links. No transition to Fickian diffusion was observed for entangled systems, even at prolonged annealing times. This suggested a higher level of kinetic restraint in entangled brush particle melts as compared to, for example, star polymers with a comparable chain length for which Fickian diffusion has been reported under similar conditions.

## Graphical abstract

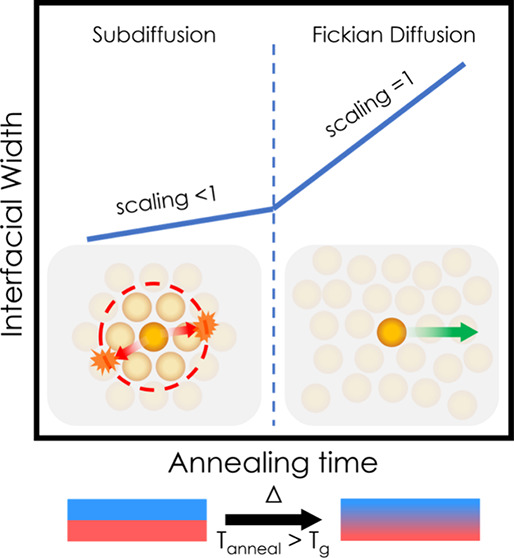

The grafting of polymeric chains to the surface of colloids, both organic and inorganic in nature, has long been used to afford uniform microstructures of particle-in-polymer dispersions.^1–4^ Advances in surface-initiated reversible deactivation radical polymerization over the past two decades now enable the deliberate control of the density, degree of polymerization, and dispersity of grafted chains – parameters that enable tailoring of the interaction, assembly formation, and properties of brush particle-based materials in the solid state.^3^ Polymer-grafted nanoparticles (aka ‘brush particles’) have thus emerged as a platform for the bottom-up fabrication of functional hybrid materials that derive unique property profiles such as increased dielectric barrier properties or thermal conductivity, selective gas transport, and impact resistance from the uniform microstructure and brush characteristics.^5–14^ More recently, brush particle solids have also been shown to feature ‘metamaterial-type’ characteristics, such as phononic hybridization gap formation – which were attributed to the heterogeneous microstructure of individual brush particles and the uniformity of their assembly structures.^15–17^ Advances in the assembly and processing of brush particle-based materials have thus fueled the vision of multifunctional metamaterial-like hybrid materials based on the deliberate assembly of ‘nanocomposite tecton’ building blocks.^18^ Harnessing these opportunities will require an understanding of not only the thermodynamic characteristics of assembly structures but also the kinetic properties governing assembly formation.

Due to the economic relevance of particle-in-polymer dispersions, most research on brush particle kinetics to date has focused on the kinetic properties of (brush) particles embedded within polymer matrices.^19^ In athermal systems (i.e., systems in which graft and matrix polymers feature equal chemical constitution), experiments and simulations revealed a nuanced dependence of the mobility of brush particles on the degree of entanglement of the matrix polymer as well as the density and wetting behavior of the brush.^20–26^ For example, simulations by Ge et al. suggested a transition from brush-dominated to particle-dominated terminal diffusion regime with increasing degree of polymerization of tethered chains.^26^ Thus, in the brush-dominated intermediate grafting regime, diffusion was found to be dominated by two subdiffusive modes related to the coupling of core as well as brush and matrix chains, whereas in the dense grafting regime hydrodynamic interactions (i.e., ‘hard sphere’ behavior) were found to be dominant.^26^ Recent molecular dynamics simulations of brush particles in entangled polymer melts confirmed an exponential slow-down of diffusivity akin to star-polymers, i.e., *D* ∼ exp­[−δ*N*_g_] (where *D* is the diffusion constant, *N*_g_ the degree of polymerization of tethered chains, and δ is a numerical constant), which was attributed to the retardation caused by the entanglement of tethered chains.^27^ Diffusion in this regime is considered to proceed by ‘arm retraction’, which was also observed in experiments by Vlassopoulos and co-workers.^28^ Interestingly, simulations also suggest a weaker dependence of *D* on *N*_g_ in the limit of high molecular brush chains due to the ‘tube renewal effect’ of the entangled polymer matrix dominating diffusion in this regime.^26^

In contrast to particle brush-in-polymer dispersions, little is known about the diffusive behavior of brush particles in the pristine melt state. This could be attributed to the limitations of experimental methods as well as the small mobility of brush particles (with a larger core size) that renders the application of in situ experiments or detailed molecular simulations difficult. In this contribution, we demonstrate the application of neutron reflectometry to determine the kinetics of layer interdiffusion in laminated brush particle bilayers. Layer interdiffusion experiments have been widely used to study the diffusive behavior of the homopolymers. The technique involves the initial fabrication of bilayer (or multilayer) films, separated by sharp interfaces, using some form of lamination technique. Subsequently, a spectroscopic technique – such as forward recoil spectroscopy or secondary ion mass spectroscopy – is used to probe the composition profile as the interface broadens during the thermal annealing of the bilayer.^29–31^ Neutron reflectometry (NR) has proven to be a particularly versatile method to quantify interdiffusion due to the nondestructive probe/sample interactions and the ability to vary the contrast between otherwise ‘chemically identical’ components by selective deuteration. Thus, the NR has been extensively used to determine the diffusivity of linear polymers from bilayer interdiffusion. For example, Russell, Karim, Kunz, Stamm, Lin and others analyzed the layer-spreading kinetics of bilayers formed by regular and deuterated polymer analogs to determine the diffusion coefficient of, for example, polystyrene (PS) or poly­(methyl methacrylate) (PMMA).^32–35^ In this contribution, we demonstrate the application of layer-spread experiments to determine the diffusion coefficient of brush particles in the melt state.

To apply the layer-spread technique to brush particle films, we synthesized organosilica (oSiO_2_)-PMMA brush particles with selectively deuterated PMMA such that the scattering length density was matched to a fully hydrogenated or deuterated oSiO_2_-core, respectively. The use of organo-modified silica (hybrid) particles was of central importance to our approach since the deuteration of the organic fraction allowed for tailoring of the scattering length density (SLD) of the particle core without affecting other properties such as grafting density or thermal stability. This allowed for the reduction of the scattering contribution of the particle cores in bilayer films and thus the quantification of interface broadening via NR. The results revealed a pronounced dependence of diffusion kinetics on the degree of polymerization (*N*_g_) of tethered chains. In the limit of low *N*_g_ (i.e., below the entanglement limit), a transition between local and Fickian diffusion was observed. In contrast, the presence of entanglements resulted in only local diffusion that was observed across all experimental time scales. The absence of Fickian diffusion across the tested time-range highlights the constraining effect imparted by entanglements in brush particle melts that exceeds those of star polymers with similar composition for which Fickian diffusion was reported under similar testing conditions.^36,37^ The results thus shed light on the physical origin of recent observations, such as shape-memory behavior or long-lived metastable states that suggest novel applications of brush particle solids.^38,39^

The synthesis of organo-silica (oSiO_2_) nanoparticles followed a process introduced by Han et al. and is illustrated in Figure 1.^40^ The reaction involved the synthesis and subsequent condensation of the organo-silica precursor 3-(triethoxysilyl)­propyl-l-α-bromoisobutyrate. Since the precursor already carried the atom transfer radical polymerization (ATRP) initiator group, the condensation resulted in ATRP initiator-functionalized oSiO_2_ particles (diameter *d*_core_ ∼ 3.3 nm) which could readily be subjected to polymer modification via SI-ATRP. Deuteration of the propyl-moiety allowed the variation of the SLD of the oSiO_2_ core across the range 1.23–3.11 × 10^10^ cm^−2^, where the limits correspond to the normal (hydrogenated) and fully deuterated form, respectively. Surface-initiated atom transfer radical polymerization (SI-ATRP) was subsequently employed to obtain PMMA grafted oSiO_2_ (oSiO_2_–PMMA) in the second step.^40–42^

**Figure 1:**
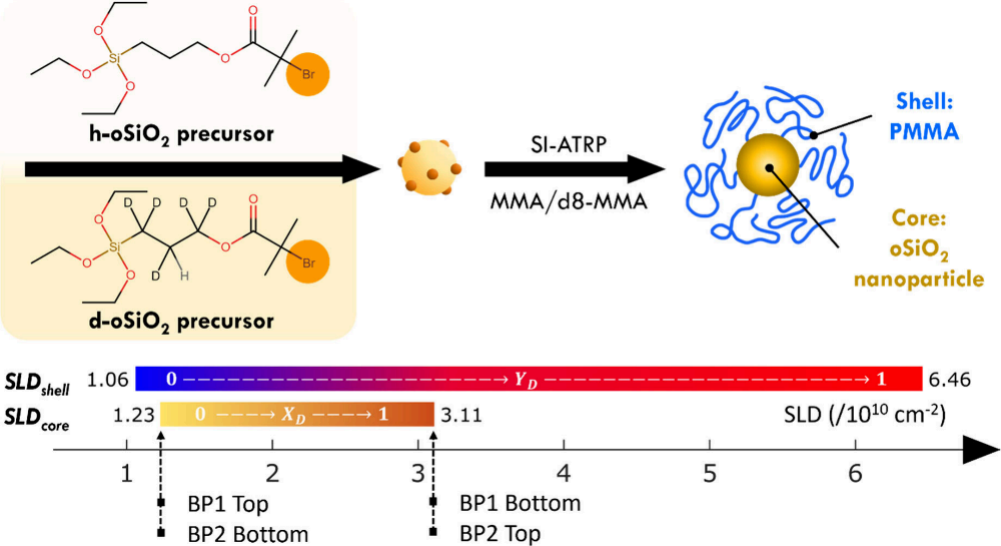
Illustration of the synthesis process of oSiO_2_ grafted onto PMMA brush particles. Tunability of the oSiO_2_ scattering length density (SLD) is accomplished by using h-oSiO_2_ and d-oSiO_2_ precursors. Active Br on the particle surface (indicated as orange spheres) acted as an initiator for grafting PMMA to obtain the brush particles. The line plot below indicates the possible SLD range of the PMMA shell and the oSiO_2_ core, respectively. SLD_shell_: 1.06–6.46 × 10^10^ cm^−2^, depending on the fraction of d8-MMA (*Y*_D_), and SLD_core_: 1.23–3.11 × 10^10^ cm^−2^, depending on the fraction of d-oSiO_2_ precursor (*X*_D_). Dotted lines indicate the residual SLDs of top and bottom layers.

Suitable stochiometric ratios of protonated MMA (h8-MMA; C_5_H_8_O_2_; SLD = 1.06 × 10^10^ cm^–2^) and deuterated MMA monomer (d8-MMA; C_5_D_8_O_2_; SLD = 6.46 × 10^10^ cm^–2^) were used for the grafting-from process to achieve the targeted shell scattering density (SLD_shell_). Here we note that SLDs were calculated with respect to the corresponding polymer form for each monomer. To match the respective SLD_core_ and SLD_shell_ of each brush particle system, while concurrently maximizing the scattering contrast between top- and bottom-layer, *X*_D_ = 0 and *Y*_D_ = 0.031 were used for the lower brush particle SLD, whereas *X*_D_ = 1 and *Y*_D_ = 0.379 were used for the higher brush particle SLD (*X*_D_ and *Y*_D_ denote the deuteration fraction of the oSiO_2_ core and PMMA grafts for each respective brush particle system). Figure 2 illustrates the resulting SLD distribution within brush particle bilayer films.

**Figure 2:**
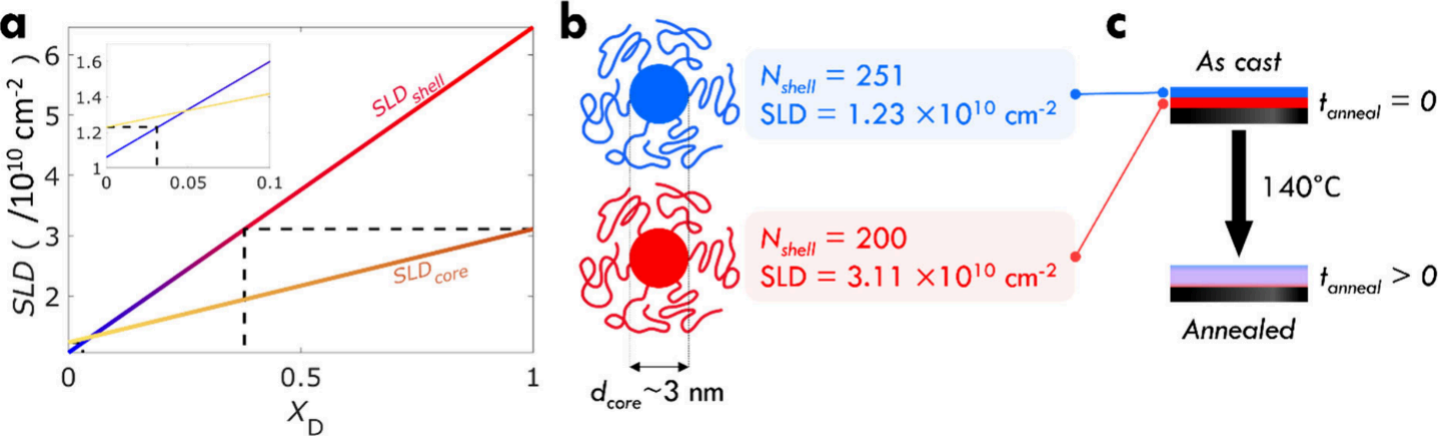
Core–shell deuteration selection and the annealing process. (a) SLD range of oSiO_2_-core and PMMA-shell depending on the deuteration fraction (*X*_D_ and *Y*_D_). Dashed lines indicate the SLD_core_ and SLD_shell_ for experiments with fully deuterated oSiO_2_. Inset shows the deuteration level for PMMA grafts on hydrogenated oSiO_2_. (b) Brush particle characteristics for layer-spread experiments. Low- and high-SLD conditions for core and shell are indicated by a blue and red color, respectively. (c) Bilayer samples with distinct SLD in each layer were prepared and then annealed for set times at 140 °C. After quenching to ambient conditions (22 °C), samples were evaluated by NR.

Linear h-PMMA and d-PMMA, with a degree of polymerization ∼ 375, were synthesized as reference polymers by regular ATRP and used for blind studies to provide validation of layer-spreading experiments. To raise the contrast, in the case of linear polymers, fully hydrogenated (h-PMMA)/deuterated (d-PMMA) compositions were chosen. The molecular characteristics of all material systems, along with their respective role in bilayer spreading experiments, are summarized in Table 1.

**Table 1:** Sample Characteristics of the Brush Particles and the Linear PMMA Used in Specular NR

				deuteration fraction		
sample ID	layering^a^	entry	*N* ^b^	oSiO_2_ (*X*_D_)	PMMA (*Y*_D_)	SLD^c^ (10^10^ cm^–2^)
BP1	top	h-oSiO_2_-(h/d)PMMA-1	252	0	0.031	1.23
	bottom	d-oSiO_2_-(h/d)PMMA-1	200	1	0.379	3.11
BP2	top	d-oSiO_2_-(h/d)PMMA-2	477	1	0.379	3.11
	bottom	h-oSiO_2_-(h/d)PMMA-2	468	0	0.031	1.23
L1	top	h-PMMA	385		0	1.06
	bottom	d-PMMA	363		1	6.46

a The stacking sequence was not expected to alter results due to the similar surface energy of h-PMMA and d-PMMA components.^43^

b Determined from the number-average molecular weight *M*_n_ from SEC.

c SLD were chosen as such to maximize the contrast. The SLD derivation of individual material (e.g., oSiO_2_ and PMMA) is shown in the Supporting Information.

For layer-spread experiments, bilayer films were prepared by individually spin-casting each layer of the film on top of separate substrates (i.e., a glass slide for the top-layer and a Si wafer for the bottom-layer) and subsequent layer lift-off and lamination. Two brush particle bilayer samples, BP1 and BP2, were prepared to investigate the effect of the degree of polymerization of the grafted chain (with ⟨*N*_g_⟩ ∼ 225 for BP1 and ⟨*N*_g_⟩ ∼ 475 for BP2). Note that the spin-casting conditions (spin rate and sample concentration in toluene) were optimized to achieve a 100 nm thickness for all films. The thickness of each layer was confirmed before and after annealing using an Alpha-Step 200 profilometer, and no changes were detected within the instrument resolution (∼5–10 nm).

For bilayer configurations of BP1 (i.e., low molecular grafted brush particles), specular NR measurements were performed after 0, 5, 10, 15, 20, 30, 45, and 120 min of thermal annealing in vacuum at 140 °C and subsequent quenching to ambient conditions (22 °C). The NR results of brush particles along with best fits to data are shown in Figure 3. Fitting of reflectivity curves was performed using ‘Refnx’, a Python package for generalized curve fitting analysis of neutron and X-ray reflectometry data.^44^ A bilayer model (i.e., h-oSiO_2_-(h/d)­PMMA and d-oSiO_2_-(h/d)­PMMA) with the addition of a native amorphous SiO_2_ layer (SLD_SiO2_ = 3.41 × 10^10^ cm^–2^) on top of the silicon substrate (SLD_Si_ = 2.07 × 10^10^ cm^–2^) were used as a fitting configuration.^45^ The best fit (based on Pearson’s χ^2^-test) showed good agreement with the NR results. The SLD profiles (i.e., SLD as a function of film height, *Z*, starting from the Si surface as *Z* = 0) obtained from the fitting process were further normalized with respect to the SLD of the higher SLD brush layer to obtain the mol-fraction profile of the higher deuteration layer (ϕ_D_), as shown in Figure 3b. The normalization was performed on the SLD profile SLD_m_(*Z*) using ϕ_D_ = (SLD_m_(*Z*) – SLD_H_)/(SLD_D_ – SLD_H_), which is easily derived from the SLD rules of mixtures, SLD_m_ = (1 – ϕ_D_)­SLD_H_ + ϕ_D_SLD_D_. Here, SLD_H_ is the SLD of the lower SLD layer (i.e., the hydrogenated layer) and SLD_D_ is SLD of the higher SLD layer (i.e., the deuterated layer). Profilometry confirmed that the thickness of the annealed samples remained about constant during annealing. A minor conditioning after onset of annealing was observed in the SLD profile (see the decrease of SLD­(*Z*) near *Z* = 0), which was attributed to the release of trapped air between the silicon/film interface. The interfacial width conjoining the two brush particle layers increased over the annealing time. The approximately stationary location of the interface between the brush particle layers at *Z* ∼ 1000 Å at different annealing times confirmed that the interdiffusion of h-oSiO_2_-(h/d)­PMMA and d-oSiO_2_-(h/d)­PMMA occurred at a similar rate (as expected based on the similar graft architectures) and that the diffusion coefficient was similar for both h-oSiO_2_-(h/d)­PMMA and d-oSiO_2_-(h/d)­PMMA.

**Figure 3:**
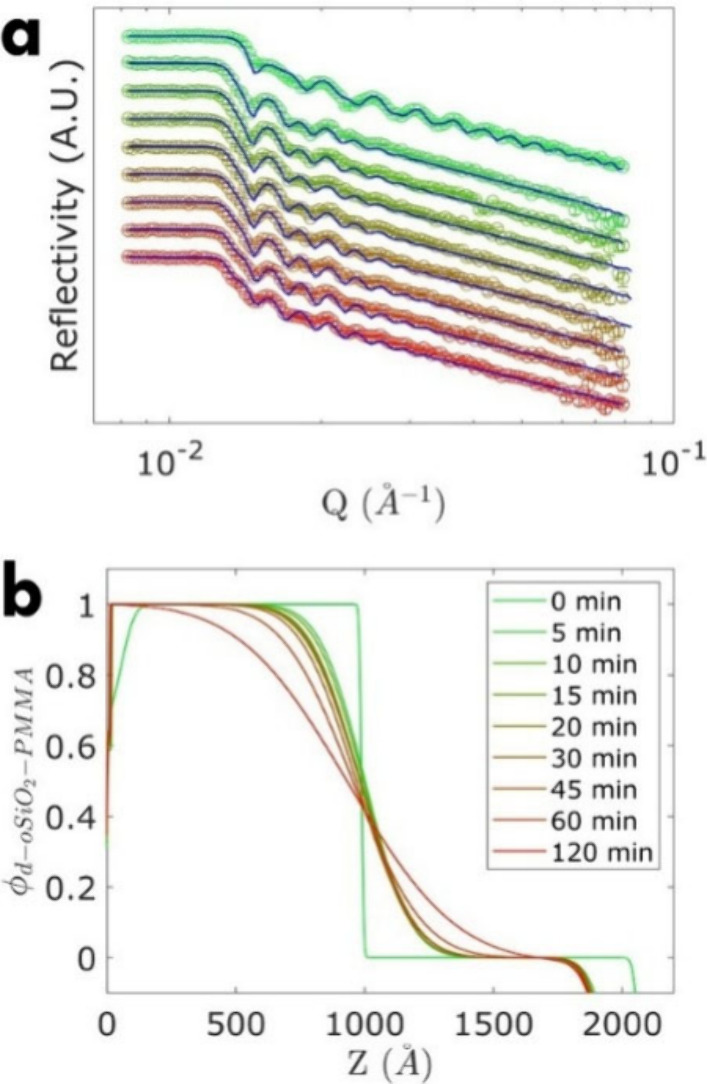
Neutron reflectometry results of the BP1 brush particle bilayer at distinct annealing times from 0 (green) to 120 min (red). (a) Specular neutron reflectivity curves along with the respective Refnx best fit; (b) Corresponding volume fraction of d-oSiO_2_-PMMA at a depth *Z* of the film.

To determine the kinetics of the brush particles, each normalized SLD profile of ϕ_D_(*Z*) was fit to the expected trend, ϕ_D_ = 0.5*c* erf­[(*h* – *Z*)/ω] + *b*, where *c* is a constant, erf­(*x*) is the error function of argument *x*, *h* is the d-oSiO_2_-PMMA layer thickness, ω is the interfacial width, and *b* is the background.^46,47^ The resulting values of ω at different annealing times were calculated, and all interfacial widths were subtracted with the initial interface width ω_0_ (=ω­(*t*_a_ = 0)) to depict the changes in interfacial width as shown in Figure 4a. The log–log plot of Δω­(*t*_a_) = ω­(*t*_a_) – ω_0_ revealed a transition in scaling coefficient (Δω ∼ *t*_a_^α^) with an annealing time at *t*_crit_ ≈ 1500 s. Two regimes could be distinguished based on the scaling coefficient. The first regime, characterized by a scaling of ∼0.13 was rationalized as the subdiffusive regime extending to time *t*_crit_. The second regime, characterized by a scaling coefficient of ∼0.5, indicated a transition to regular Fickian (Brownian) diffusion at *t*_a_ > *t*_crit_. The diffusion coefficient (*D*) was subsequently determined from the data within the Fickian’s regime via (Δω)^2^ = 4*Dt*_a_, as shown in Figure 4b, and the result revealed the (self-) diffusion coefficient of oSiO_2_-PMMA as *D*_BP1_ = 6.87 × 10^–16^ cm^2^/s.

**Figure 4:**
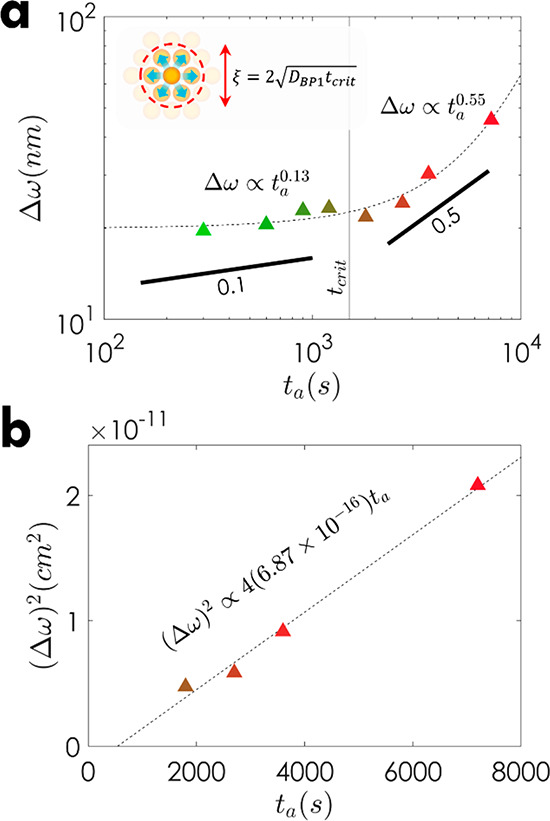
Interfacial width analysis used to determine the diffusion coefficient of the oSiO_2_-PMMA brush particles. (a) Log–log plot of the substracted interfacial width, Δω, as a function of annealing time, *t*_a_, to identify the respective diffusion regime. The dashed lines are included to guide the eye, and a solid vertical line at *t*_crit_ ≈ 1500 s shows the approximate transition time between the two regimes. The inset depicts the estimated cage size of ξ ∼ 3*d* which the particle needs to escape to transition to the Fickian regime (*d* is the brush particle diameter). (b) Plot of ω^2^ as a function of *t*_a_ for all results above the transition time. The resulting diffusivity was determined using *D*_BP1_ = (Δω)^2^/(4*t*_a_) = 6.87 × 10^–16^ cm^2^/s.

To validate the procedure, the interdiffusion of a bilayer configuration of linear h-/d-PMMA was analyzed under equivalent conditions (Figure S1). The analysis revealed a diffusion coefficient for PMMA with ⟨*N*⟩ ∼ 375 at 140 °C of *D*_PMMA_ = 1.74 × 10^–15^ cm^2^/s, close to the values reported in the literature.^34^ Interestingly, analogous NR experiments on the higher molecular brush particle systems BP2 (⟨*N*⟩ ∼ 470, Figure S2) revealed no transition to the Fickian regime. Instead, the kinetics were dominated by the subdiffusive regime as indicated by the scaling Δω ∼ *t*_a_^0.37^ for all tested time scales (Figure S3).

To interpret the interdiffusion results for BP1 and BP2, we first compare *D*_BP1_ with the estimated diffusion coefficient of linear PMMA of equivalent molecular weight. To estimate the total brush particle size, we used the established two-layer model (TLM), which yielded for the brush height *h* = (*R* ^3^ + (3*R* ^2^σ*N*_k_)/ρ_PMMA_)^1/3^ – *R* = 1.86 nm (where *R* is the particle radius).^49^ The total brush particle size could therefore be estimated as *d* ≈ 7 nm. This matches approximately the total brush particle size estimated from TEM micrographs and dynamic light scattering (DLS) (Figure S4). Hence, for a linear PMMA with equivalent radius of gyration, *R*_g_, the molecular weight can be estimated to be ∼51300 g/mol.^46^ Since for (entangled) linear polymer melts the diffusivity and molecular weight have been shown to be related by *D* ∼ *M* ^–2.30^, it followed that the diffusion coefficient of such a linear polymer should be about 3.35 × 10^–16^ cm^2^/s, about equal to *D*_BP1_.^48^ The similarity of both values was surprising, since arm-entanglement was expected to result in an exponential slow-down of diffusivity.^36,37^ We attributed the absence of a star polymer-type retardation to the low molecular weight of grafted chains (⟨*N*_g_⟩ ∼ 220), which was just below the commonly reported threshold for entanglement formation of PMMA.^49^

To test for entanglement formation in BP1 and BP2, films deposited on Cu grids (i.e., TEM sample holders) were subjected to bend deformation to induce fracture and craze formation. The latter is commonly regarded as evidence for entanglement formation in amorphous polymers.^50^ Figure 5 displays TEMs of crack tip regions in monolayer films of the pristine constituent brush materials of systems BP1 and BP2, respectively. The figure revealed rather sharp fracture surfaces for brush particle systems comprising BP1 (Figure 5a,b), whereas fibrils connecting fracture surfaces (i.e., crazes) could be observed for both brush systems comprising BP2 (Figure 5c,d).

**Figure 5:**
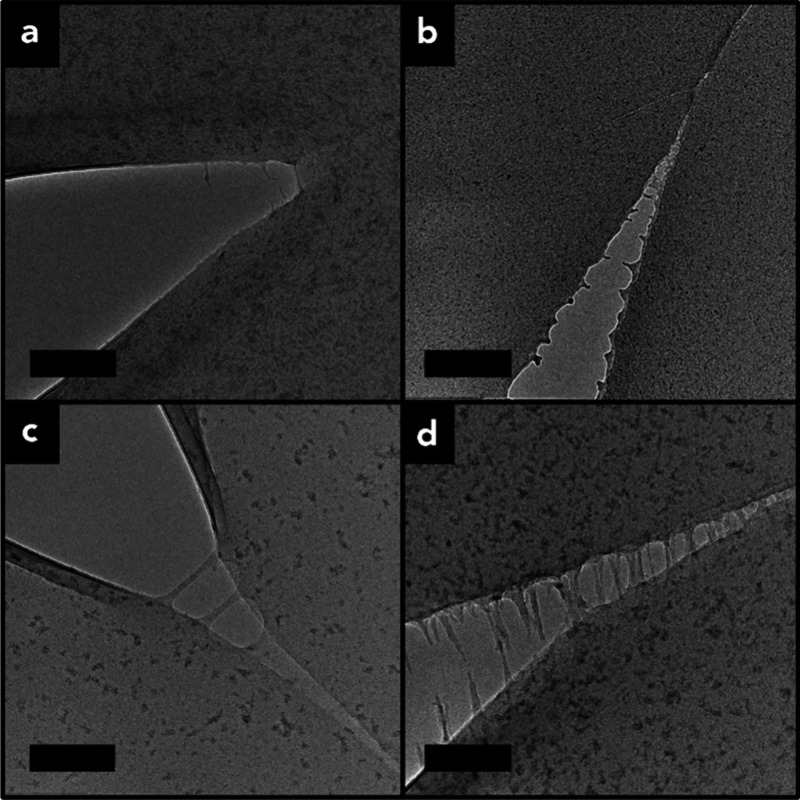
Crack characteristics in oSiO_2_ brush particle films: (a) h-oSiO_2_-(h/d)­PMMA-200, (b) d-oSiO_2_-(h/d)­PMMA-252, (c) h-oSiO_2_-(h/d)­PMMA-468, and (d) d-oSiO_2_-(h/d)­PMMA-477. Craze formation in panels (c) and (d) supports entanglement formation in brush particle materials comprising system BP2. Scale bars: 200 nm.

Thus, the observed transition to Fickian diffusion for BP1, with a diffusion constant like that of a linear polymer of equivalent molecular weight, was attributed to the absence of entanglement formation. To further interpret the role of the characteristic time scale *t*_crit_ marking the transition between sub- and Fickian diffusion, we note that the average distance traversed by a brush particle during a time interval *t*_crit_ is about (4*Dt*_crit_)^1/2^ ∼ 22.2 nm, i.e., approximately equal to three times the size of a brush particle. This corresponds to the estimated effective size of a coordination sphere formed by nearest neighbors in brush particle melts (see the inset in Figure 4a). This suggests that *t*_crit_ could be interpreted as a characteristic ‘caging time’, i.e., the time needed for a brush particle to escape the cage formed by its nearest neighbors. Thus, we hypothesize that the absence of entanglements in BP1 rendered the diffusion similar to the cage dynamics which was observed previously in hard sphere-type colloidal suspensions rather than star-polymer analogs.^51,52^

The absence of a Fickian diffusion regime for BP2 across the experimental time range (Figure S3) was surprising since experiments on star-polymers with similar number and length of arms (albeit at somewhat larger *T* – *T*_g_), revealed Fickian diffusion even across shorter time ranges. For example, Shull et al. reported a systematic evaluation of the kinetics of polystyrene (PS) star polymers with number of arms ranging 3–12 using forward recoil spectrometry.^36^ For a five-arm star PS (i.e., comparable to the number of grafts per oSiO_2_–PMMA) with degree of polymerization *N*_g_ ∼ 530, the diffusion coefficient at 178 °C was reported to be *D* ∼ 10^–15^ cm^2^/s. Interestingly, the size of the impenetrable inner core of the stars studied by Shull and co-workers could be estimated using the Daoud-Cotton model, as *d*_core_ = 2*f* ^1/2^ *l* ∼ 3 nm (where *f* is the number of arms and *l* = 0.67 nm is the statistical segment length of PS), i.e., close to the oSiO_2_ core size of 3.5 nm.^53,54^ Considering the similar core size dimensions, the absence of a Fickian regime in BP2 across an even wider experimental time range is surprising and could be attributed, for example, to the stiffer (i.e., larger elastic modulus) core composition that conceivably could amplify the retardation effect due to graft entanglements and hence impart stronger constraints on core mobility. The results thus illuminate the possible physical origin of long-lived metastable states that have been reported for brush particle solids, such as the stabilization of surface patterns or the display of shape memory properties in brush particle solids.^39,55,56^

In conclusion, the selective deuteration of organosilica brush particles enabled the measurement of brush particle diffusion coefficients by layer interdiffusion using neutron reflectivity. The diffusion coefficient sensitively depended on the degree of polymerization of the tethered chains. Below the entanglement limit, a transition from sub- to Fickian diffusion was observed, suggesting a ‘cage diffusion-like’ mechanism similar to granular and concentrated colloidal systems.^57,58^ Thus, brush particle diffusion in such systems involved the temporary trapping and localized motion of particle cores within transient ‘coordination spheres’ formed by the cores of adjacent brush particles in the melt state. Increasing the molecular weight beyond the entanglement limit rendered subdiffusion the dominant diffusion mode across the entire experimental time range. The absence of Fickian diffusion (across the tested range) indicated a significant extension of the escape time, presumably due to the additional constraints caused by graft entanglements or the stiffness of the particle cores. The flexibility of the organosilica platform should render a more detailed analysis of particle core size, grafting density, or dispersity of tethered chains straightforward extensions of the present work. Further, the extension to all-organic particle core compositions, such as those introduced by Yin et al., could provide a path to understand the role of ‘stiffness’ on the strength of constraints implied by particle cores.^59^

## Supplementary Material



## Notes

SLD calculation; NR results of linear PMMA; NR results for BP2 brush particle bilayer; TEM and DLS results of BP1 (PDF)
