# Supplementary material for: Neutron Reflectometry Reveals Diffusion in Contrast-Matched Brush Particle Bilayers
Source: ACS Macro Lett. 2026 Apr 6;15(4):633–9. doi: 10.1021/acsmacrolett.6c00109 (PMC13104167; doi:10.1021/acsmacrolett.6c00109)
Supplement: Supplementary file 1 [file mz6c00109_si_001.pdf]

Supporting Information for Publication

Neutron Reflectometry Reveals Diffusion in Contrast-Matched Brush Particle Bilayers

Jirameth Tarnsangpradit,[†] Yuqi Zhao,[†] Hanshu Wu,[‡] Rongguan Yin,[‡] Hanyu Wang,[ⱡ] Akhtar Gul,[§] Alamgir Karim,[§] Krzysztof Matyjaszewski,[‡] Michael R. Bockstaller[†]*

[†] Department of Material Science and Engineering, Carnegie Mellon University, 5000 Forbes Avenue, Pittsburgh, Pennsylvania 15213, United States

[‡] Department of Chemistry, Carnegie Mellon University, 4400 Fifth Avenue, Pittsburgh, Pennsylvania 15213, United States

[ⱡ] Center for Nanophase Materials Sciences, Oak Ridge National Laboratory, Oak Ridge, Tennessee 37831, United States

[§] Department of Chemical and Biomolecular Engineering, University of Houston, Houston, Texas 77204, United States

* Correspondence author: bockstaller@cmu.edu

1. SLD Calculation

The neutron scattering length density (SLD) of linear PMMA (h-PMMA and d-PMMA) were determined from SLD = $(\rho N_{Av}/m)\ \Sigma n_i b_i$ where ρ is the mass density (~1.18 g/cm^3 for PMMA and d8-PMMA), N_{Av} is the Avogadro's number, m is the repeat unit molecular weight of a polymer (100.1 g/mol for PMMA and 108.2 for d8-PMMA), $\Sigma n_i b_i$ is the total scattering length by summing the scattering length of atom i (b_i) with n_i atoms within the repeat unit. The scattering length data can be referred to from literature [1]. The SLDs of the calculated h-PMMA and d-PMMA shown in **Table1** are also consistent with literature [2].

Similar to the SLD calculation of PMMA, the atomic composition and the mass density are needed for the SLD calculation of the organosilica nanoparticle core. From **Figure1**, the atomic composition of h-$oSiO_2$ precursor is $Si(OC_2H_5)_3C_7H_{12}O_2Br$ (and is $Si(OC_2H_5)_3C_7D_5H_7O_2Br$ for d-$oSiO_2$ precursor). To form the core initiator, the precursors were condensed to form crosslinked through Si-O-Si, meaning each of the oxygen atoms connected to silicon atoms are shared between adjacent precursors. Hence, the atomic composition becomes $C_7H_{12}O_{2+1.5}SiBr$ for h-$oSiO_2$ (and $C_7D_5H_7O_{3.5}SiBr$ for d-$oSiO_2$-Br). As for the mass density, a value of 1.61 g/cm^3 was used based on previous literature [3].

2. Neutron Reflectometry Results of Linear PMMA

The neutron reflectometry results along with the fittings and the reduced SLD profile of linear PMMA with estimates degree of polymerization of 375. The annealing was performed at 140 °C with different annealing times from 0 to 15 minutes. Note that the reduced SLD profiles were calculated by normalizing the SLD profile with high SLD layer (e.g., 6.46×10^{10} cm^{-2} for fully deuterated PMMA or d-PMMA) which gives the volume fraction of high SLD layer shown in **Figure S1b**.

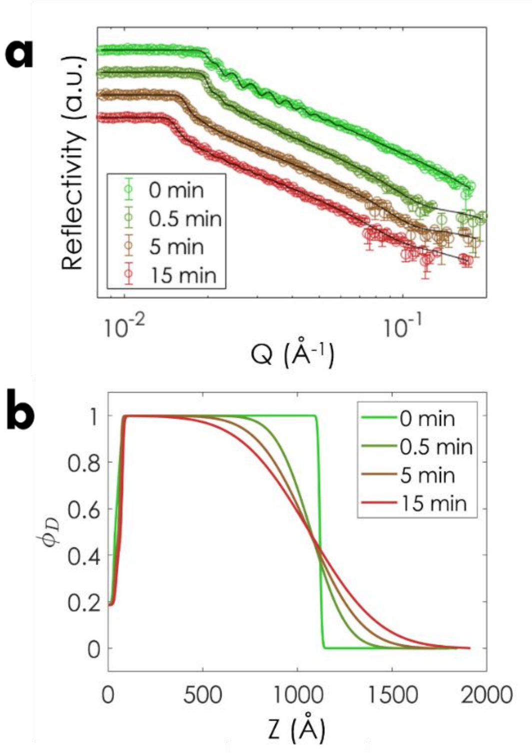

Figure S1. Neutron reflectometry results of linear PMMA (N = 375) at distinct annealing times from 0 minute (green) to 15 minutes (red). (a) depicts specular neutron reflectometry curves along with the respective Refnx best fit; (b) depicts the volume fraction d-PMMA at depth Z of the film.

3. Neutron Reflectometry Results of higher molecular weight brush particles: BP2

The neutron reflectivity results and SLD profiles of the organosilica-grafted-PMMA brush particles, BP2, at different annealing time varying from 0 to 45 minutes with 5 minutes increment. Note that the reflectivity curves are shifted for visuals. The SLD profiles (**Fig. S2b**) show consistent interface location over time, suggesting that the diffusion rate of both layers is similar. **Figure S3** shows the interfacial width plot over annealing time. The resulting scaling of 0.37 reveals BP2 to have subdiffusion behavior for the obtained experimental time.

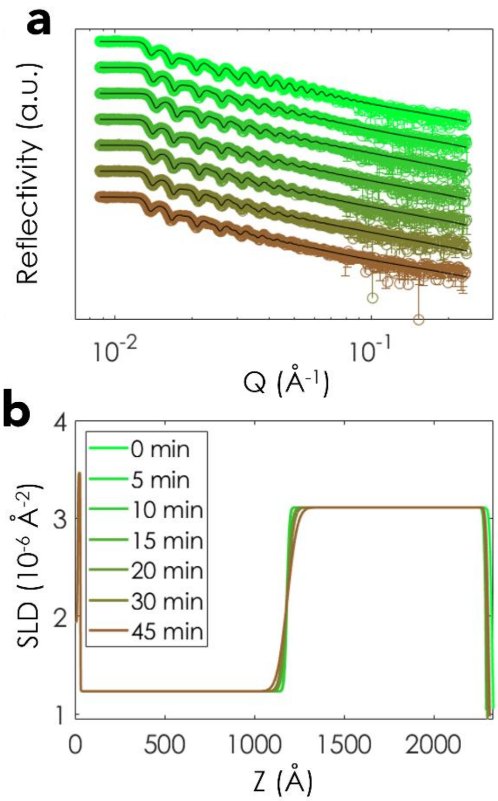

Figure S2. NR results and SLD profile of BP2 ($N \sim 475$) with annealing temperature at 140 °C.

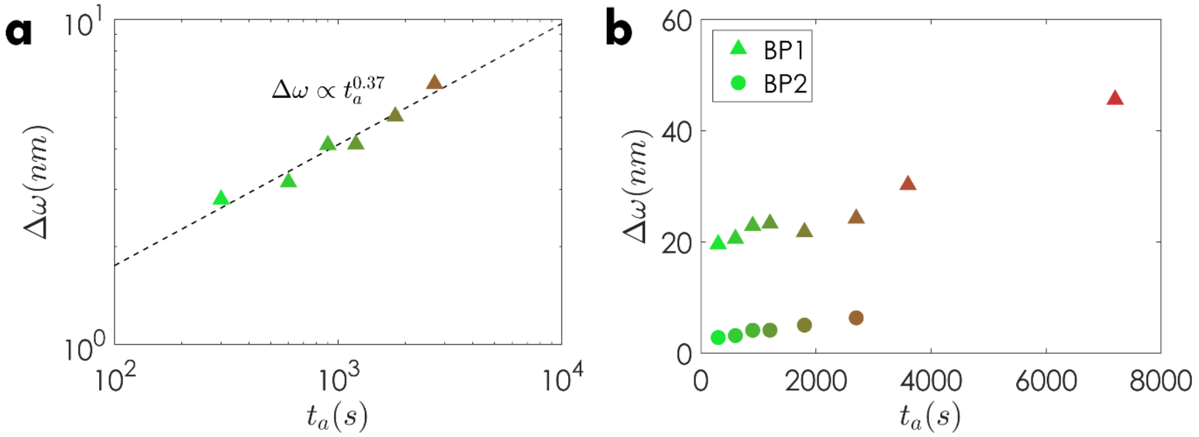

Figure S3. (a) Interfacial width results of BP2 revealing scaling of 0.37. (b) Comparison of the interfacial width between BP1 and BP2.

4. TEM and DLS revealing size of the brush particle BP1

The transmission electron micrograph along with the dynamic light scattering (DLS) of the brush particle BP1 is shown below in **Figure S4**. This image along with other TEM micrographs were obtained using a FEI Tecnai F20 TEM/STEM at 200 kV.

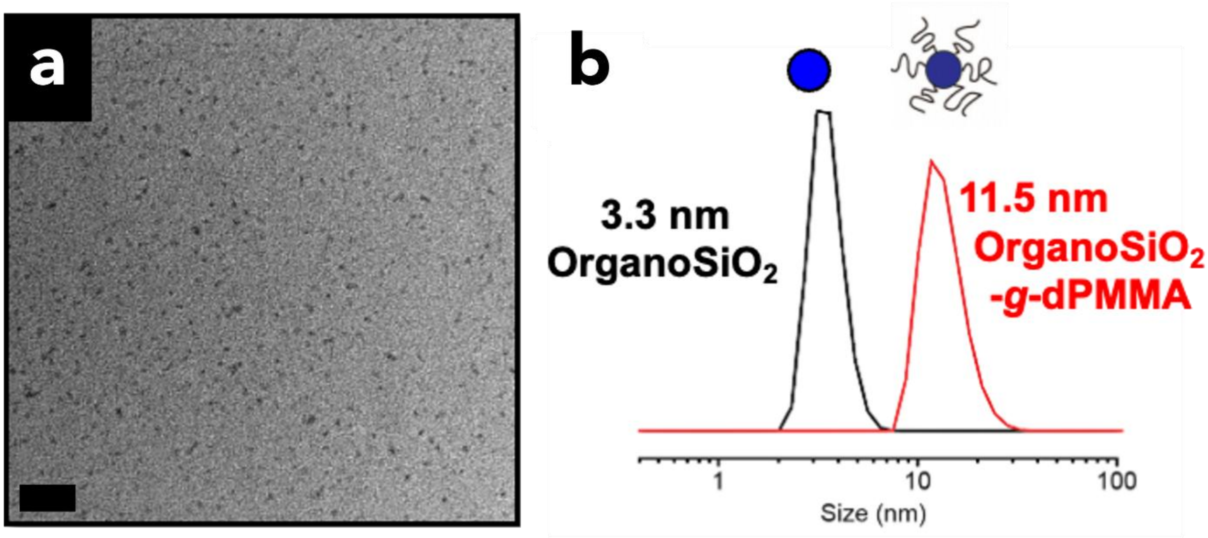

Figure S4. (a) TEM and (b) DLS results of h-SiO_2-(h/d)PMMA-200 brush particles. Note that the scale bar shown is 50 nm and the measured core size is 3.3±0.5 nm.

References

(1) Sears, V. F. Neutron Scattering Lengths and Cross Sections. *Neutron News* **1992**, *3* (3), 26–37. https://doi.org/10.1080/10448639208218770.

(2) Ooe, M.; Miyata, K.; Yoshioka, J.; Fukao, K.; Nemoto, F.; Yamada, N. L. Direct Observation of Mobility of Thin Polymer Layers via Asymmetric Interdiffusion Using Neutron Reflectivity Measurements. *J. Chem. Phys.* **2019**, *151* (24), 244905. https://doi.org/10.1063/1.5132768.

(3) Han, J.; Zhai, Y.; Wang, Z.; Bleuel, M.; Liu, T.; Yin, R.; Wu, W.; Hakem, I. F.; Karim, A.; Matyjaszewski, K.; Bockstaller, M. R. Nanosized Organo-Silica Particles with "Built-In" Surface-Initiated Atom Transfer Radical Polymerization Capability as a Platform for Brush Particle Synthesis. *ACS Macro Lett.* **2020**, *9* (9), 1218–1223. https://doi.org/10.1021/acsmacrolett.0c00502.
